# Coal Home Heating and Environmental Tobacco Smoke in Relation to Lower Respiratory Illness in Czech Children, from Birth to 3 Years of Age

**DOI:** 10.1289/ehp.8501

**Published:** 2006-02-28

**Authors:** Rebecca J. Baker (posthumous), Irva Hertz-Picciotto, Miroslav Dostál, Jean A. Keller, Jiři Nožička, František Kotìšovec, Jan Dejmek (posthumous), Dana Loomis, Radim J. Šrám

**Affiliations:** 1 Department of Epidemiology, School of Public Health, University of North Carolina–Chapel Hill, Chapel Hill, North Carolina, USA; 2 Division of Epidemiology, Department of Public Health Sciences, School of Medicine, University of California–Davis, Davis, California, USA; 3 Institute of Experimental Medicine, Academy of Sciences, Prague, Czech Republic; 4 Regional Institute of Hygiene, Prachatice, Czech Republic; 5 Health Institute of Central Bohemia, Prague, Czech Republic; 6 Department of Environmental Sciences and Engineering, University of North Carolina–Chapel Hill, Chapel Hill, North Carolina, USA

**Keywords:** air pollution, breast-feeding, bronchitis, children’s health, coal heating, environmental tobacco smoke, indoor

## Abstract

**Objective:**

The objective of this study was to evaluate how indoor pollution from tobacco and home heating may adversely affect respiratory health in young children.

**Design:**

A birth cohort was followed longitudinally for 3 years to determine incidence of lower respiratory illness (LRI).

**Participants:**

A total of 452 children born 1994–1996 in two districts in the Czech Republic participated.

**Evaluations:**

Indoor combustion exposures were home heating and cooking fuel, mother’s smoking during pregnancy, and other adult smokers in the household. Diagnoses of LRI (primarily acute bronchitis) from birth to 3 years of age were abstracted from pediatric records. Questionnaires completed at delivery and at 3-year follow-up provided covariate information. LRI incidence rates were modeled with generalized linear models adjusting for repeated measures and for numerous potential confounders.

**Results:**

LRI diagnoses occurred more frequently in children from homes heated by coal [vs. other energy sources or distant furnaces; rate ratio (RR) = 1.45; 95% confidence interval (CI), 1.07–1.97]. Maternal prenatal smoking and other adult smokers also increased LRI rates (respectively: RR = 1.48; 95% CI, 1.10–2.01; and RR = 1.29; 95% CI, 1.01–1.65). Cooking fuels (primarily electricity, natural gas, or propane) were not associated with LRI incidence. For children never breast-fed, coal home heating and mother’s smoking conferred substantially greater risks: RR = 2.77 (95% CI, 1.45–5.27) and RR = 2.52 (95% CI, 1.31–4.85), respectively.

**Conclusions:**

Maternal smoking and coal home heating increased risk for LRI in the first 3 years of life, particularly in children not breast-fed.

**Relevance:**

Few studies have described effects of coal heating fuel on children’s health in a Western country. Breast-feeding may attenuate adverse effects of prenatal and childhood exposures to combustion products.

Children < 3 years of age, and especially in their first year, are at greatest risk of serious respiratory illnesses (Phelan et al. 1994). Studies from industrialized areas indicate that the rate of childhood lower respiratory illness (LRI) peaks during the first year of life. Globally, particularly in developing countries, acute lower respiratory infections are the most important cause of death among children < 5 years of age, accounting for about two million deaths per year (Bruce et al. 2000).

Recently, increasing attention has focused on effects of indoor air exposures on respiratory health of children (Bruce et al. 2002; Chen et al. 1990; Ezzati and Kammen 2002; Honicky and Osborne 1991; Marbury et al. 1996; Smith et al. 2000). These exposures include combustion products; semivolatile and volatile organic compounds released by furnishings, building materials, and chemical products; pollutants from volatilization of chemicals in soil; and pollutants generated by decomposition of organic matter (Smith et al. 2000). The main indoor pollutants from combustion are carbon monoxide, nitrogen oxides, sulfurous oxides, particles, and volatile organics. In developing countries, early childhood respiratory illness has been associated with use of biomass or coal as fuel for heating and cooking (Bruce et al. 2002; Ezzati and Kammen 2002; Smith et al. 2000). In more developed countries, reports on respiratory health effects of combustion emissions from wood (Honicky and Osborne 1991; Honicky et al. 1983; Tuthill 1984) and natural gas (Braun-Fahrlander et al. 1992; Farrow et al. 1997; Samet et al. 1993) in the home have been mixed, whereas recent studies examining coal home heating have found increased reports of respiratory symptoms such as phlegm and cough in adults (Pope and Xu 1993) and school children (Jedrychowski et al. 1998; Qian et al. 2004a, 2004b). Few studies, if any, have been conducted in Western countries using physician diagnoses for respiratory illnesses (other than asthma) in early childhood. Associations between environmental tobacco smoke (ETS) and children’s respiratory illnesses are well established (Li et al. 1999; Strachan and Cook 1998).

Heightened susceptibility of infants and young children to environmental toxicants has been suggested. Inhaled pollutant dose per unit body weight is likely to be greater, because their body weight is smaller and their respiratory rate higher than in adults (Cerna et al. 1998; Gilliland et al. 1999). Infants’ developing lungs and immune system put them at greater risk for respiratory infections and may make them particularly vulnerable to inhaled pollutants’ irritative and immune effects (Koenig 2000; Phelan et al. 1994), especially in the first year of life while the lungs are still maturing (Braga et al. 2001). In air pollution studies, infant mortality is associated with ambient particle concentrations (Bobak and Leon 1999; Loomis et al. 1999; Penna and Duchiade 1991; Saldiva et al. 1994; Woodruff et al. 1997). Breast-feeding protects against respiratory infections, particularly in the first year of life (Heinig 2001; Phelan et al. 1994). Breast-feeding also may modify adverse effects of ETS exposure on respiratory illnesses (Chulada et al. 2003; Nafstad and Jaakkola 2003; Nafstad et al. 1996; Woodward et al. 1990).

This study, part of a large program of research on air pollution health effects in the Czech Republic (Teplice Program), focused on respiratory illnesses in young children in relation to indoor combustion of cigarettes and of coal, wood, natural gas, and propane for heating or cooking. A high proportion of adults smoke in the Czech Republic, and coal is still used for heating of some homes; hence, this setting is advantageous for studying indoor air exposures.

## Materials and Methods

### Study population

We followed a birth cohort to 3 years of age in two districts in the Czech Republic: Teplice in the northwest, and Prachatice in the southwest. The Teplice district, with a population of about 130,000, is infamous for high air pollution due to power plants and home heating with coal. The Prachatice district has a population of about 50,000 and no power plants. Children born between May 1994 and December 1996 in the two districts were eligible for this study.

**Figure 1:**
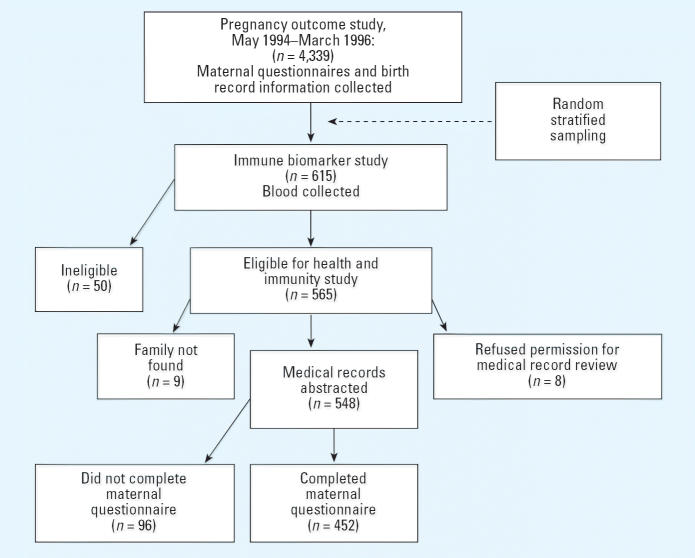
Evolution of study sample, beginning with the POS, which enrolled close to 90% of eligible births. A stratified random sample was selected for the IBS. The final sample for this study consisted of children with pediatric records and maternal follow-up questionnaires.

The evolution of our study sample is shown in Figure 1. As part of the Teplice program (Šrám et al. 1996), Dejmek et al. (1999, 2000a, 2000b, 2002) enrolled 4,339 mother–infant pairs at birth into a study of pregnancy outcomes and air pollution, the pregnancy outcome study (POS). Only singleton births were included. A probability sample of that group, with higher fractions of low-birth-weight (< 2,500 g) and preterm (< 37 completed weeks of gestation) infants, was enrolled in the immune biomarker study (IBS; *n* = 615) (Hertz-Picciotto et al. 2002). Initially, investigators randomly sampled every fifth normal-birth-weight infant and all low-birth-weight infants; however, sampling fractions were increased in Prachatice because of a lower birth rate than expected, and in Teplice to enroll more children starting in January 1996 during a meteorologic inversion. All sampling was random within strata. Infants born on Fridays and Saturdays were ineligible for the IBS, because immunologic analyses had to be performed within 24 hr of blood draw.

For this investigation, we recontacted the IBS participants at 3 years of age. Of the 615 IBS children, 50 were ineligible for follow-up (32 families had moved to another district, 11 children were adopted or in social care, and 7 children died). Of the remaining 565, pediatric data were abstracted for 548 (97%): Nine families were not located for follow-up, and eight mothers denied permission to review medical records. Of the 548 with pediatric data available, 452 (82.5%) of the mothers completed a 3-year follow-up questionnaire. Their children were the subjects of this analysis. This study was approved by the institutional committees on human subjects at the Institute for Experimental Medicine in Prague, the University of North Carolina, Chapel Hill, and the University of California, Davis.

### Data collection

Trained nurses administered questionnaires at the birth hospital within 3 days of delivery and elicited reproductive and medical history; medications; demographic information; smoking, alcohol, and other lifestyle factors; and work histories and occupational exposures. For the 3-year follow-up, pediatric nurses contacted families to invite their participation and provided them a new questionnaire regarding breast-feeding, child care attendance, child’s and family members’ allergies, indoor heating and cooking fuel sources, and information about household members’ age, relation to child, and smoking behaviors. Informed consent was administered at birth and again at follow-up, before collection of data.

Physicians or nurses abstracted medical data from birth and pediatric records. At birth, information collected included mother’s obstetric, labor, and delivery complications; newborn sex, birth weight, Apgar scores, and congenital anomalies; and clinicians’ estimate of gestational age. When the child was 3 years of age, health providers abstracted dates of all visits, diagnoses using the *International Classification of Diseases, 10th Revision* (ICD-10) codes (World Health Organization 1993), and any treatments or medications prescribed. The use of a standard pediatric medical record form throughout the country facilitated transfer of information onto the study forms. Records of visits to specialists, hospitals, or emergency services are forwarded to the primary physician, so these reports were also abstracted and included in the analyses. When an ICD-10 code was not provided on our study form, medically trained study personnel coded the diagnosis using the medical chart notes. Because medical care is free and universally available, pediatrician utilization is high, and most families stay with the same pediatrician until their children reach maturity.

### LRI events

The 452 children in our study sample experienced 1,049 LRI events based on ICD-10 codes J20, J21, J40, and J44, of which 98% were acute bronchitis (J20). Pneumonia diagnoses (*n* = 70) were excluded from this analysis. The date of pediatrician’s diagnosis served as a proxy for date of illness occurrence. To avoid counting the same illness twice, we did not include diagnoses occurring within 30 days after a previous LRI diagnosis, leaving 893 incident LRI events.

### Indoor combustion emissions exposures

Mothers reported indoor heating and cooking fuel combustion sources, such as gas-, wood-, or coal-burning devices in the home on the 3-year follow-up questionnaire. We initially analyzed incidence of LRI within categories of heating and cooking fuel and by presence of gas-using appliances (stove, oven, and water heater) in the home. Because most fuels for heating conferred no increase in LRI rates or were used by very few households, in final models we compared coal home heating with all other heating sources. Direct measurements of organic carbon, elemental carbon, or other elements were not made in homes.

Information on ETS exposure was available from both the birth and 3-year questionnaires. We assessed mother’s smoking during pregnancy (yes/no) and adult smokers in the household at 3-year follow-up (yes/no).

### Covariates

Covariates of interest were selected *a priori* based on a review of the literature and preliminary bivariate analyses. Time-independent covariates included district of residence (Teplice vs. Prachatice), child’s sex, maternal ethnicity [either Central European or Rom (“Gypsy”) based on self-report], and maternal education (categorized as 6–10 years, 11 years, and ≥ 12 years for tabular presentation, and coded as a continuous variable in final models).

On the 3-year follow-up questionnaire, mothers were asked until what age their child was “fully” and “partially” breast-fed; we used the maximum of these two responses to construct three time-varying categories: currently breast-feeding, ever breast-fed, and never breast-fed. We also constructed categories for time since cessation of breast-feeding, to capture possible protection beyond the weaning period: currently breast-feeding, breast-fed in the last month, last breast-fed 1–6 months ago, last breast-fed > 6 months ago, and never breast-fed. For analyses of breast-feeding as a modifier of the effects of indoor air exposures, we dichotomized children as ever or never breast-fed.

Other time-dependent variables included child’s age in years, current child care attendance (yes/no, based on maternal report of the ages at which the child attended child care with other children), number of other household members ≤ 14 years of age (0, 1, and ≥ 2), and household density (number of adults and children living in the household divided by the number of rooms). The seasons were winter (December–February), spring (March–May), summer (June–August), and autumn (September–November). This categorization captured school vacation (June–August) as a distinct period. Days of the week were grouped based on similarity in LRI rates: Saturday–Sunday, Monday, and Tuesday–Friday.

We examined temperature and relative humidity using 24-hr means from which we calculated moving averages for 3, 7, 14, and 30 days before (and including) the day of diagnosis. Of these averaging periods, 14-day average temperature showed the strongest association with LRI and therefore was selected. The log-odds of LRI decreased linearly with temperature, permitting a continuous term for temperature. Relative humidity was not associated with LRI diagnosis.

### Statistical methods

We used generalized estimating equations (GEEs) to model the associations with LRI diagnoses (Zeger and Liang 1986; Zeger et al. 1988). This method provides robust variance estimates, which account for the correlation among repeated observations in the same individuals (Stokes et al. 2000). GEEs also allow characterization of effects from time-varying factors (listed above). Data were structured in a child-day file, where each row corresponded to one observation-day for one child. A child followed for exactly 3 years would contribute 1,095 –30*n* observation-days, where *n* is the number of LRI events separated by at least 30 days. On a given day of life, occurrence of an LRI was coded 1; nonevent days were coded 0.

We used a logit link, that is, a logistic model, of the binary outcome (event day: yes/ no) and specified an exchangeable correlation structure to account for greater within-child than between-children homogeneity. The probability (rate per child-day) of an LRI event occurring on any given child-day was small (0.002). Odds of an event are therefore arithmetically very close to the per child-day rate. The rate ratio (RR) was estimated by *e*^β^ for each variable, or category, and corresponding 95% confidence intervals (CIs) were calculated.

Because the children in this follow-up study were not a simple random sample of births in Teplice and Prachatice districts, we also accounted for the unequal sampling probabilities of normal-birth-weight/full-term and low-birth-weight/preterm infants, which differed by district and by year of study. Inverse sampling probabilities were used as weights with a design of stratified sampling without replacement. Model fitting was conducted using SUDAAN software (version 8.0; Research Triangle Institute, Research Triangle Park, NC, USA).

Initially, we determined “crude” rates of LRI (LRI event counts per child-time at risk) within categories of predictor variables. RRs and 95% CIs were then estimated using GEE models adjusted for sampling design, but not for other covariates. Next, covariates associated (defined loosely as Wald chi-squared *p*-value of < 0.15) with LRI in bivariable analyses were entered into a full model, and less influential covariates were removed, one by one, beginning with the least influential (greatest *p*-value). Covariates for which removal changed the point estimates for coal heat or ETS by ≥ 10% were retained as confounders. Final models included coal heating, mother’s smoking during pregnancy, presence of other adult smokers in the household, mother’s age and education, child’s sex and year of life, breast-feeding, child care attendance, number of other children living in the home, season, day of the week, and 14-day moving-average temperature.

We examined potential modification of the indoor pollutant–LRI associations by year of life, breast-feeding, preterm birth and/or low birth weight, child’s sex, child care attendance, temperature, and season by inclusion of product terms with ETS or coal home heating, one at a time. We also explored variability of effects by year of life in three separate models, to evaluate more closely factors that changed considerably over the first 3 years (e.g., breast-feeding and child care attendance).

## Results

Fifty percent of homes were heated primarily by distant heating (heat from a remote source outside the home), 23% by natural gas, 13% by coal, and 6% by wood (Table 1). Natural gas, propane, or electricity accounted for 97% of primary cooking fuels used; only 3% of families reported cooking primarily with wood or coal. Twenty-four percent of mothers reported smoking during pregnancy, and 50% of fathers smoked at time of delivery. At 3-year follow-up, 35% of mothers reported smoking; almost 60% of children lived in homes with at least one smoker; and 28% of homes had two or more smokers.

Eight percent of mothers were of Rom ethnicity, and 87% breast-fed the children, 23% for > 6 months. Twenty-one percent of children attended child care with other children at some time during the first 3 years of life, and about three-fifths lived in households with another child.

Coal heat, mother’s smoking during pregnancy, and ETS during the first 3 years of life were all associated with greater incidence of LRI (Table 2). Use of a wood- or gas-burning stove or other appliance in the home did not increase LRI rates significantly. Other factors associated with 40–50% higher rates of LRI were Rom ethnicity, current attendance at child care with other children, and presence of other children in the household. Mondays and winter months were also associated with greater numbers of LRI diagnoses. Protective factors included increasing maternal age, maternal education, and temperature (each with a monotonic relationship), as well as breast-feeding. Low birth weight or preterm birth did not affect LRI rates.

The observed associations between indoor combustion exposures and LRI incidence persisted after multivariate control for mother’s age and education, child’s sex and year of life, breast-feeding, current attendance at child care, other children living in the household, season, day of the week, and 14-day moving-average temperature (Table 3). Children living in homes where coal was used as the primary heating fuel experienced 45% greater LRI incidence (RR = 1.45; 95% CI, 1.07–1.97) compared with children whose homes were heated by natural gas, propane, electricity, wood, or a distant (outside the home) source. Mother’s smoking during pregnancy increased her child’s LRI incidence over the next 3 years by 48% (RR = 1.48; 95% CI, 1.10–2.01), and an adult other than the mother smoking in the household independently increased child’s LRI incidence 29% (RR = 1.29; 95% CI, 1.01–1.65). Among children never breast-fed, the effects of both coal home heating (RR = 2.77; 95% CI, 1.45–5.27) and mother’s smoking (RR = 2.52; 95% CI, 1.31–4.85) on LRI incidence were greater than among children who were breast-fed (Table 4). The never–breast-fed group included more Romany, more mothers with higher education, and more smokers. There was some suggestion that the coal heating effect was primarily in the first 2 years of life (data not shown), but no other effect modification was as striking as that of breast-feeding.

## Discussion

We found that children exposed to indoor coal combustion experience a greater incidence of pediatrician-diagnosed LRI during the first 3 years of life. We did not observe associations between LRI incidence and use of natural gas or propane as heating fuel. The coefficient for wood as a heating source was elevated, but the CIs were wide, largely because wood was used by very few households. Coal, wood, and propane for cooking showed elevated but imprecise relative risks, and both coal and wood were rarely used for cooking. Fewer than half of families with gas heating had a furnace inside the flat, suggesting that exposures such as nitric oxide and nitrogen dioxide would occur only in some of the homes. Nevertheless, previous studies of infants also indicate no increases in LRI from exposures to nitrogen dioxide (Samet et al. 1993). Thus, our data were consistent with findings of no increased risk associated with emissions from gas cooking and heating.

Indoor measurements were not available. The coal burning devices are stand-alone units located inside the living spaces of the homes, which either directly heat the air or heat water that circulates through a radiator. Furnaces located outside the homes were designated as “distant sources” and included in the reference group. Although the venting is always to the outside, indoor air becomes polluted with dust and gases when coal is added or the unit is cleaned by removal of the ashes. Nearby outdoor air is also polluted by normal operation of these units. Studies of emissions sources conducted in Teplice and Prachatice in the early 1990s showed that home heating with lignite coal contributed measurably to outdoor concentrations of ambient organic and elemental carbon, sulfur, potassium, iron, zinc, lead, bromine, and other elements (Pinto et al. 1998). Moreover, it was shown that during the winter, the ratio of benzo[*a*]pyrene to lead in ambient outdoor air increased 5–15 times over the ratio observed in summer months, which was attributed to emissions from residential home heating by coal combustion (Stevens et al. 1997). In houses occupied by nonsmokers, major sources of indoor pollution are nearby homes that use coal for fuel (Benes et al. 2003). Thus, emissions vented outside may make their way back into the flat through windows and doors. Given the climate in these districts (mean daily temperatures < 10°C, or 50°F, for more than half the year), homes would be heated on a daily basis for not less than 6 months a year. Indoor environments are likely to be heavily polluted in homes where coal is used for fuel.

Higher vulnerability of very young children to indoor pollutants may occur for several reasons. Infants and toddlers spend more time indoors at home than do school-age children or adults (Brinkman et al. 1999; U.S. Environmental Protection Agency 2002; Wiley et al. 1991). Indoor air exposures can be more concentrated than outdoor exposures, especially when sources are indoors and buildings are closed, as in the winter. Additionally, the respiratory and immune systems are undergoing development during early life: In this study population, district of residence, season, and prenatal exposures to ambient pollution were related to altered distributions of lymphocyte immunophenotypes and IgE in cord blood (Hertz-Picciotto et al. 2002), with some data implicating specific vulnerable time periods (Herr CEW, unpublished data; Hertz-Picciotto et al. 2005).

Our findings for both maternal smoking during pregnancy and postnatal ETS from other household smokers are similar to results of meta-analyses (Li et al. 1999; Strachan and Cook 1997). The risk of developing an acute LRI in the first 3 years of life is increased about 60% if either parent smokes, 70% if the mother smokes, and about 30% if the mother does not smoke but other household members do (Strachan and Cook 1997). Moreover, we found independent effects on young children’s LRI risk from mothers’ smoking during pregnancy and from postnatal ETS due to other adults’ smoking. Previous work suggests that ETS carries the greatest risk to children < 3 years of age (Kontos et al. 1999). Because of the strong correlation between maternal smoking during and after pregnancy, we could not evaluate which time period contributed most to the effects we observed.

Among children who had never breast-fed, associations with both coal combustion and mother’s smoking were substantially greater, with LRI rates elevated close to 3-fold and 2.5-fold, respectively. Breast-feeding has been found to protect against the effects of maternal smoking on LRI in infants (Nafstad et al. 1996; Woodward et al. 1990) and older children (Chulada et al. 2003). Coal heating emissions and ETS share similar pollutant constituents, so it was not surprising to find that breast-feeding protected children from the adverse effects of coal home heating. Immunity conferred by breast milk is understood to be both anti-infectious and anti-inflammatory (Hanson et al. 2001, 2002). Breast-feeding may play a direct protective role against indoor combustion exposures, and it may foster key immunologic responses, thereby reducing susceptibility to infection.

Our study relied on home environmental data collected retrospectively. Some error may be introduced when mothers are asked to recall events over the past 3 years (e.g., when she stopped breast-feeding or when the child began child care). These errors seem unlikely to differ by exposure, but could possibly mask covariate–respiratory illness associations, which may limit the ability to control for these covariates as potential confounders of pollutant–respiratory illness associations.

Another limitation was the lack of information on changes in some home environmental characteristics between birth and 3 years of age. At the time of our study, a national policy was in effect to replace coal in home heating with natural gas, with the goal to decrease outdoor air pollution. Despite this, about 13% of the study children’s homes were still heated primarily by coal at follow-up (1997–1999). A family that switched from coal to gas would have reported use of gas at 3-year follow-up, resulting in misclassification of heating type for some part of the 3-year follow-up. Generally, this might have attenuated the results, with some of the “unexposed” children actually exposed to coal. If switching were related to other factors, the direction of bias would be difficult to predict.

This study used pediatrician-reported LRI. Capture of an LRI diagnosis depends on health service utilization, as well as the ability of the pediatrician to reliably record the type of illness. Our data demonstrate extensive utilization of health care services by this population of Czech mothers. Despite some changes in the provision of health care in the Czech Republic over the past decade, including the emergence of private health insurance, mostly provided by employers, all residents are entitled to health care, and consumer cost is relatively low. An indication of the high utilization of physicians is the completeness of the legally mandated 18-and 36-month pediatric well-child visits, which in our study sample was > 96% and > 98%, respectively. Rates of complete series of immunizations for diphtheria, pertussis, and tetanus (DPT); polio; and measles were high, far exceeding U.S. 1997 rates for children of the same age (Centers for Disease Control and Prevention 2001). For example, 98% of children in our cohort received a complete series of four DPT immunizations, compared with 71% of children this age in the United States in 1997 (Centers for Disease Control and Prevention 2001). With regard to reliable recording of events, we surveyed most of the pediatricians who participated in this study about how they coded children with specific sets of symptoms and found that their responses were highly consistent and similar across the two districts, and matched expected ICD-10 codes for overall LRI. Because virtually all previous studies of coal home heating used parental reports of illnesses or symptoms, sometimes for retrospective recall over long periods, the present study represents a considerable improvement in the quality of health outcome data.

## Conclusions

In summary, we found exposure to coal home heating and ETS increase young children’s LRI rates during the first 3 years of life. These effects were attenuated by breast-feeding. Although ETS has been well studied, residential coal combustion in economically developed countries has not; these findings demonstrate that both sources of indoor air combustion pollutants present a hazard to respiratory health in infancy and early childhood. Efforts to reduce these emissions would benefit infants and young children perhaps especially in the Czech setting, where coal is still commonly used in home heating and smoking rates are relatively high.

## Figures and Tables

**Table 1:** Characteristics of the study sample, Teplice and Prachatice districts (*n* = 452).

Characteristic	No. (%)
Home heating	
Outside the flat (distant heating)	224 (49.6)
Natural gas	106 (23.5)
Electricity	34 (7.5)
Coal	59 (13.1)
Wood	26 (5.8)
Unknown/other	3 (0.7)
Primary fuel used for cooking	
Gas	192 (42.5)
Propane	54 (12.0)
Electricity	194 (42.9)
Coal	4 (0.9)
Wood	8 (1.8)
Mother smoked during pregnancy	
Yes	109 (24.1)
No	343 (75.9)
Mother smoked at 3-year follow-up	
Yes	159 (35.2)
No	284 (62.8)
Unknown	9 (2.0)
Father smoked at time of delivery	
Yes	231 (51.1)
No	216 (47.8)
Unknown	5 (1.1)
No. of adult smokers living in the home	
0	187 (41.4)
1	137 (30.3)
2	107 (23.7)
≥ 3	18 (4.0)
Unknown	3 (0.7)
District of residence	
Teplice	265 (58.6)
Prachatice	187 (41.4)
Mother’s age at delivery (years)	
< 20	44 (9.7)
20–24.9	219 (48.5)
25–29.9	122 (27.0)
30–34.9	52 (11.5)
≥ 35	15 (3.3)
Mother’s ethnicity	
Czech/European	415 (91.8)
Rom	37 (8.2)
Mother’s education (years)	
6–10	91 (20.1)
11	166 (36.7)
≥ 12	193 (42.7)
Unknown	2 (0.4)
Father’s education (years)	
6–10	75 (16.6)
11	181 (40.0)
≥ 12	190 (42.0)
Unknown	6 (1.3)
Child’s sex	
Male	250 (55.3)
Female	202 (44.7)
Gestational age (weeks)	
< 37	41 (9.1)
≥ 37	411 (90.9)
Birth weight (g)	
1,500–2,499	41 (9.1)
2,500–3,499	238 (52.7)
3,500–4,499	173 (38.3)
Year of birth	
1994	76 (16.8)
1995	142 (31.4)
1996	234 (51.8)
Duration of breast-feeding (months)	
0	57 (12.6)
1–3	196 (43.4)
4–6	91 (20.1)
7–12	70 (15.5)
> 12	35 (7.7)
Unknown	3 (0.7)
Attended child care between 0 and 3 years of age	
Yes	93 (20.6)
No	345 (76.3)
Unknown	14 (3.1)
No. of other children ≤ 14 years of age in the home	
0	191 (42.3)
1	203 (44.9)
≥ 2	55 (12.2)
Unknown	3 (0.7)
Household density	
0.00–0.99	48 (10.6)
1.00–1.99	305 (67.5)
≥ 2.00	96 (21.2)
Unknown	3 (0.7)

a RRs and 95% CIs are estimated using GEE models and are adjusted for both sampling design and repeated events.

b Assessed at 3-year follow-up.

c Assessed at time of child’s birth.

**Table 2:** Bivariable analysis of LRIs in relation to covariates.

Covariate/category	No. of events	No. of child-months	Rate per child-month	RR (95% CI)^a^
Heating fuel^b^				
Distance heat + other	427	7,803	0.055	1.00
Natural gas	198	3,680	0.054	1.01 (0.73–1.39)
Electricity	59	1,184	0.050	0.92 (0.52–1.65)
Coal	154	2,007	0.077	1.56 (1.13–2.15)
Wood	50	901	0.055	1.18 (0.66–2.10)
Cooking fuel^b^				
Electricity	389	6,708	0.058	1.00
Gas	351	6,672	0.053	0.92 (0.70–1.22)
Propane	125	1,852	0.068	1.30 (0.89–1.91)
Coal	11	135	0.081	1.29 (0.26–6.45)
Wood	17	276	0.062	1.20 (0.72–1.99)
Average no. of cigarettes mother smoked per day during pregnancy^c^				
0	591	11,953	0.049	1.00
1–5	174	2,389	0.073	1.79 (1.32–2.43)
6–10	95	1,040	0.091	1.88 (1.22–2.89)
> 10	33	260	0.127	2.30 (1.02–5.17)
Total no. of family members who smoke (including mother)^b^				
0	282	6,557	0.043	1.00
1	314	4,700	0.067	1.61 (1.20–2.18)
≥ 2	294	4,280	0.07	1.76 (1.29–2.41)
Total no. of family members who smoke (excluding mother)^b^				
0	381	7,850	0.049	1.00
1	452	6,975	0.065	1.33 (1.02–1.74)
≥ 2	57	711	0.080	1.54 (0.87–2.75)
District				
Teplice	508	9,185	0.055	0.97 (0.75–1.25)
Prachatice	385	6,457	0.060	1.00
Mother’s age (years)^c^				
< 20	87	1,523	0.057	1.13 (0.76–1.66)
20–24.9	455	7,557	0.060	1.00
25–29.9	254	4,209	0.060	0.94 (0.71–1.25)
30–34.9	79	1,824	0.043	0.71 (0.41–1.21)
≥ 35	18	531	0.034	0.59 (0.35–1.00)
Mother’s ethnicity				
Czech	797	14,383	0.055	1.00
Rom	96	1,259	0.076	1.49 (1.04–2.12)
Mother’s education (years)^c^				
6–10	237	3,095	0.077	2.05 (1.51–2.80)
11	370	5,706	0.065	1.60 (1.21–2.12)
≥ 12	282	6,773	0.042	1.00
Father’s education (years)^c^				
6–10	169	2,575	0.066	1.43 (0.99–2.07)
11	417	6,207	0.067	1.29 (0.95–1.76)
≥ 12	294	6,654	0.044	1.00
Child’s sex				
Male	579	8,572	0.068	1.50 (1.16–1.94)
Female	314	7,071	0.044	1.00
Preterm (< 37 weeks gestation) and/or low birth weight (< 2,500 g)				
Yes	123	2,182	0.056	1.00 (0.72–1.40)
No	770	13,460	0.057	1.00
Child’s year of life				
First	326	5,200	0.063	1.32 (1.06–1.63)
Second	299	5,205	0.057	1.19 (0.99–1.42)
Third	268	5,238	0.051	1.00
Breast-feeding				
Current	92	2,183	0.042	0.51 (0.33–0.78)
0–1 month ago	19	372	0.051	0.83 (0.43–1.60)
1–6 months ago	127	1,835	0.069	1.05 (0.69–1.59)
> 6 months ago	509	9,201	0.055	0.77 (0.52–1.13)
Never	139	1,948	0.071	1.00
Currently attending child care with other children				
Yes	66	856	0.077	1.43 (0.93–2.19)
No	804	14,291	0.056	1.00
No. of other children ≤ 14 years of age in the home^b^				
0	330	6,658	0.050	1.00
1	442	6,984	0.063	1.30 (0.98–1.74)
≥ 2	118	1,894	0.062	1.14 (0.80–1.62)
Household density^b^				
0–0.99	67	1,688	0.040	0.78 (0.48–1.26)
1–1.99	585	1,057	0.055	1.00
≥ 2	238	3,276	0.073	1.29 (0.96–1.73)
Season				
Winter	333	3,748	0.089	3.20 (2.49–4.12)
Spring	223	3,916	0.057	2.29 (1.78–2.95)
Summer	117	4,037	0.029	1.00
Fall	220	3,942	0.056	2.06 (1.57–2.71)
Day of the week				
Saturday–Sunday	85	4467.43	0.019	0.29 (0.21–0.40)
Monday	229	2234.17	0.102	1.63 (1.40–1.91)
Tuesday–Friday	579	8941.03	0.065	1.00
14-Day moving-average temperature (°C)				
< 0	247	2,278	0.108	2.86 (2.36–3.46)
0–9.9	383	5,678	0.067	1.81 (1.49–2.19)
10–19.9	234	6,402	0.037	1.00
≥ 20	22	1,041	0.021	0.65 (0.39–1.09)

a RRs and 95% CIs are estimated using GEE models and are adjusted for both sampling design and repeated events.

b Assessed at 3-year follow-up.

c Assessed at time of child’s birth.

**Table 3:** Estimated RRs and 95% CIs from full model predicting LRI among children from birth to 3 years of age, Teplice and Prachatice districts, 1994–1999 (*n* = 452).

Parameter	RR (95% CI)^a^
Indoor combustion exposures	
Heating fuel	
Coal heating	1.45 (1.07–1.97)
All others^b^	1.00
ETS	
Mother smokes^c^
Yes	1.48 (1.10–2.01)
No	1.00
Adult other than mother smokes^d^	
Yes	1.29 (1.01–1.65)
No	1.00
Covariates	
Mother’s age (years)	
< 20	1.04 (0.80–1.36)
20–29.9	1.00
≥30	0.52 (0.25–1.07)
Mother’s education (per year of education)	0.93 (0.85–1.00)
Child’s sex	
Male	1.29 (1.01–1.65)
Female	1.00
Year of life	
First	1.53 (1.14–2.05)
Second	1.19 (0.99–1.44)
Third	1.00
Breast-feeding	
Currently	0.47 (0.29–0.76)
Weaned 0–1 month ago	0.68 (0.33–1.43)
Weaned 1–6 months ago	1.05 (0.68–1.60)
Weaned > 6 months ago	0.97 (0.65–1.43)
Never breast-fed	1.00
Child care with other children	
Currently attending	1.42 (0.92–2.18)
Never attended	1.00
No. of other children living in the home	
0	1.00
1	1.24 (0.93–1.64)
≥2	0.88 (0.60–1.28)
Season	
Fall	1.25 (0.90–1.75)
Winter	1.12 (0.74–1.70)
Spring	1.37 (1.01–1.84)
Summer	1.00
Day of the week	
Saturday–Sunday	0.28 (0.21–0.39)
Monday	1.68 (1.43–1.97)
Tuesday–Friday	1.00
Temperature	
14-Day moving average (per °C)	0.95 (0.93–0.96)

a Adjusted for all other variables in the table, as well as both sampling design and repeated events, using GEEs in logistic regression models.

b Includes distant heating and use of natural gas, electricity, or wood for heat in the home.

c Used mother’s response to smoking during pregnancy from questionnaire administered at delivery.

d Used report of household members’ smoking behavior at 3-year follow-up.

**Table 4:** Adjusted RRs and 95% CIs,^a^ relating coal home heating and mother’s smoking to incidence of LRI, by breast-feeding status over the first 3 years of life, Teplice and Prachatice districts, 1994–1999 (*n* = 452).

Exposure	RR (95% CI)
Coal home heat	
Ever breast-fed	1.33 (0.95–1.86)
Never breast-fed	2.77 (1.45–5.27)
Mother smokes	
Ever breast-fed	1.37 (0.98–1.92)
Never breast-fed	2.52 (1.31–4.85)

a RRs and 95% CIs estimated from GEE model results, adjusted for sampling design, other adults’ smoking, mother’s age, child’s sex and year of life, child care attendance, siblings, season, day of the week, and 14-day average temperature.
